# High-fat diet promotes colitis-associated tumorigenesis by altering gut microbial butyrate metabolism

**DOI:** 10.7150/ijbs.86717

**Published:** 2023-09-25

**Authors:** Xinyu Shao, Luojie Liu, Yuqing Zhou, Kaiqiang Zhong, Jinrong Gu, Tong Hu, Yizhou Yao, Chunli Zhou, Weichang Chen

**Affiliations:** 1 Department of Gastroenterology, The First Affiliated Hospital of Soochow University, Suzhou 215006, Jiangsu, China.; 2 Department of Gastroenterology, The Affiliated Suzhou Hospital of Nanjing Medical University, Suzhou Municipal Hospital, Gusu School, Nanjing Medical University, Suzhou 215008, Jiangsu, China.; 3 Department of Gastroenterology, Changshu Hospital Affiliated to Soochow University, Suzhou 215000, Jiangsu, China.; 4 Department of General Surgery, The First Affiliated Hospital of Soochow University, Suzhou 215006, Jiangsu, China.

**Keywords:** High fat diet, colitis associated cancer, gut microbiota, butyrate metabolism, macrophage polarization

## Abstract

**Background:** Dietary fat intake is associated with an increased risk of colitis associated cancer (CAC). A high-fat diet (HFD) leads to systemic low-grade inflammation. The colon is believed to be the first organ suffering from inflammation caused by the infiltration of pro-inflammatory macrophages, and promotes CAC progression. We explored the role of HFD in driving CAC by altering gut microbial butyrate metabolism.

**Methods:** Changes in the gut microbiota caused by HFD were investigated via HFD treatment or fecal microbiota transplantation (FMT). The underlying mechanisms were further explored by analyzing the role of gut microbiota, microbial butyrate metabolism, and NLRP3 inflammasome in colon tissues in a CAC mouse model.

**Results:** HFD accelerated CAC progression in mice, and it could be reversed by broad-spectrum antibiotics (ABX). 16S-rRNA sequencing revealed that HFD inhibited the abundance of butyrate-producing bacteria in the gut. The level of short-chain fatty acids (SCFAs), especially butyrate, in the gut of mice treated with HFD was significantly reduced. In addition, treatment with exogenous butyrate reversed the M1 polarization of proinflammatory macrophages, aggravation of intestinal inflammation, and accelerated tumor growth induced by HFD; the NLRP3/Caspase-1 pathway activated by HFD in the colon was also significantly inhibited. *In vitro*, macrophages were treated with lipopolysaccharide combined with butyrate to detect the M1 polarization level and NLRP3/Caspase-1 pathway expression, and the results were consistent with those of the *in vivo* experiments.

**Conclusion:** HFD drives colitis-associated tumorigenesis by inducing gut microbial dysbiosis and inhibiting butyrate metabolism to skew macrophage polarization. Exogenous butyrate is a feasible new treatment strategy for CAC, and has good prospect for clinical application.

## Introduction

The consumption of a high-fat diet (HFD) induces chronic low-level inflammation, associated with various metabolic and inflammation-related diseases 1, 2. Long-term HFD and related chronic inflammation disrupt intestinal immune homeostasis. Epidemiological studies have revealed that excessive HFD intake is closely associated with the progression and recurrence of inflammatory bowel disease (IBD) 3, 4. Inflammation is one of the widely recognized carcinogenic pathways. The intestinal mucosa of patients with IBD, especially ulcerative colitis (UC), is at high risk of developing colitis-associated cancer (CAC) because of long-term exposure to inflammation. Therefore, it is generally considered that prolonged IBD is a precancerous lesion of CAC, and the cancer occurrence rate in patients with IBD over 10 years is higher than 10% 5.

With the advent of genome sequencing and microbiome identification technology, the gut microbiota diversity is gradually being explored 6, 7. The important roles of gut microbiota include regulating the immune inflammatory response of the host intestine, synthesizing various small molecules and proteins, and regulating the absorption of nutrition 8, 9. Small molecule metabolites transmit chemical signals, which are a conventional way of communication between bacteria and bacteria and between bacteria and intestinal mucosa 10-12. The intestinal epithelium forms a barrier between the body and the contents of the intestinal cavity by a single layer of epithelial cells. The intestinal immune system constantly maintains a delicate balance between tolerance toward symbiotic bacteria and immunity against pathogenic bacteria, and an imbalance in this is one of the important factors leading to excessive intestinal inflammatory response 13-15.

As a part of the intestinal immune system, macrophages play an important role in removing pathogens, regulating inflammation, and maintaining local homeostasis 16, 17. Macrophage M1 polarization secretes TNF-α, IL-6, IL-1β, and other pro-inflammatory cytokines; produces nitric oxide; and participates in inflammatory response. In contrast, macrophage M2 polarization generally produces anti-inflammatory factors, such as IL-10 and TGF-β, which promote tissue repair and remodeling 18-20. However, whether macrophage M1/M2 polarization can remain balanced after long-term HFD consumption, the factors driving macrophage activation, and whether macrophage M1/M2 polarization imbalance is involved in the process of CAC remain unclear. Our study intends to deeply analyze the effect and mechanism of HFD in IBD patients, and provide a new treatment strategy and theoretical basis for the intervention of gut microbiota in IBD patients with long-term HFD exposure.

## Materials and Methods

### Establishment of the CAC mouse model

Specific pathogen-free (SPF) grade male C57BL/6 mice (aged 4-6 weeks, Shanghai Institutes for Biological Sciences) were fed sterile food and water and maintained in sterile isolators. The mice were randomly assigned to groups. To meet the 3R principle and for the calculation of sample size, an appropriate sample size was determined according to a previous study using C57BL/6 mice 21-23. Ciprofloxacin (0.2g/L) and metronidazole (1g/L) were added to drinking water for intestinal pretreatment for 2 weeks. Subsequently, the mice were intraperitoneally injected with azoxymethane (AOM, 10 mg/kg, Sigma Aldrich), a colorectal cancer mutagen. The drinking water of the mice was supplemented with 2.5% dextran sulfate sodium (DSS, Sigma Aldrich) on Day 1, Day 21, and Day 41 for five consecutive days and then changed to normal water.

Our study design is illustrated based on the CAC mouse model, and comprises three parts 24. In the first part, while establishing the mouse CAC model, the mice were divided into three groups including control diet (CD), high-fat diet (HFD), and HFD combined with broad-spectrum antibiotics (ABX, vancomycin 500 mg/L, imipenem 500 mg/L, and neomycin 1 g/L) groups. In the second part, after pretreatment with antibiotics, the mice were divided into CD-fecal microbiota transplantation (FMT) and HFD-FMT groups for establishing the CAC model. FMT was performed with fecal samples from CD group and HFD group, respectively, three times a week until the end of the experiment. In the third part, after pretreatment with antibiotics, HFD-FMT was performed and mice were divided into butyrate treatment and water treatment groups. The mice in the butyrate treatment group were given drinking water containing 2mg/mL sodium butyrate (Sigma, USA) until the end of the experiment.

The colorectal polyps of mice were evaluated on Day 60, and weight change, colon length, spleen weight, number of polyps, and polyp size were measured. Tissue samples were frozen in liquid nitrogen or fixed in 10% formalin for further analysis. The entire colon was stained with hematoxylin and eosin (H&E). Colon inflammation, area, hyperplasia, and dysplasia of the mucosa and submucosa, and the pathological factors were observed by H&E staining. The results were evaluated by two authors who were blinded to each other 25.

### Immunofluorescence staining

The colon tissues were embedded in paraffin and 5-μm sections were cut. They were incubated with indicated primary antibodies followed by Alexa Fluor 488-conjugated secondary antibodies for immunofluorescence staining. The slides were then washed three times and stained with DAPI (4',6-diamidino-2-phenylindole). Images were taken with a Nikon ECLIPSE Ni microscope equipped with a color camera and were processed by ImageJ software. Five randomly selected regions were analyzed per sample.

### 16S rRNA sequencing

The 16S rRNA sequencing community map was identified by the Illumina HiSeq sequencing V4 region (insertion size of 300 bp; read length of 250 bp). Sequences were reaggregated with 97% sequence consistency, and chimeras were removed by UPARSE. For each representative sequence, the GreenGene database was used to label the classification information. Changes in bacterial abundance were analyzed, and the bacteria with significant differences were selected as candidates.

### Fatty acid detection by GC-MS

Each sample (50 mg) was mixed with 2 mL of 1% sulfuric acid-methanol solution, mixed thoroughly for 1 min, and placed in a water bath at 80℃. After 30 min of methylation, 1 mL of n-hexane was added for extraction, and the sample was washed with 5 mL of pure water. The supernatant (500 μL) was then added to 100 mg of anhydrous sodium sulfate to remove excess water and an internal label was added to the supernatant for testing. After gas chromatography-mass spectroscopy (GC-MS) detection, the samples were quantitatively analyzed by referring to the standard curve.

### Detection of macrophage cell activation

The *RAW264.7* macrophage cell line (from the American Type Culture Collection) was treated for 24 h with butyrate (1 mM). The expression of PE-inducible nitric oxide synthase (INOS) was detected by flow cytometry to assess M1 macrophage polarization. *RAW264.7* cells were also treated for 24 h with a combination of butyrate (1 mM), adenosine triphosphate (ATP, 2 mM), and lipopolysaccharide (LPS, 10 ng/mL). Subsequently, M1 polarization was investigated. The cell lines used in this study were previously authenticated.

### Protein extraction and Western blot analysis

Total proteins were separated by sodium dodecyl sulfate-polyacrylamide gel electrophoresis (SDS-PAGE) and transferred onto polyvinylidene fluoride (PVDF) membranes, which were blocked with 5% non-fat milk and sequentially incubated with the indicated primary antibodies and horseradish peroxidase-conjugated secondary antibodies. The proteins were visualized by chemiluminescence and the signals were quantified by ImageJ software.

### Ethics statement

This study was approved by the Institutional Ethics Committee of the First Affiliated Hospital of Soochow University (reference number: 2022337) and the Affiliated Suzhou Hospital of Nanjing Medical University (reference number: K-2021-077). All animal experimental procedures were approved by the Animal Ethics Committee of the First Affiliated Hospital of Soochow University and the Affiliated Suzhou Hospital of Nanjing Medical University according to the ARRIVE guidelines 2.0.

### Statistical methods

All data are presented as mean ± SEM of at least three independent experiments. P < 0.05 was considered statistically significant. Statistical analyses were performed using SPSS 25.0 software (SPSS Inc., Chicago, IL, USA), GraphPad Prism 8 (San Diego, CA), and R programs (https://cran.r-project.org/web/packages.html). The student's t-test (unpaired, two-tailed), Mann-Whitney U test, or one-way ANOVA were used to compare means between groups.

## Results

### HFD accelerates CAC depending on gut microbiota and related metabolites

**Figure 1:**
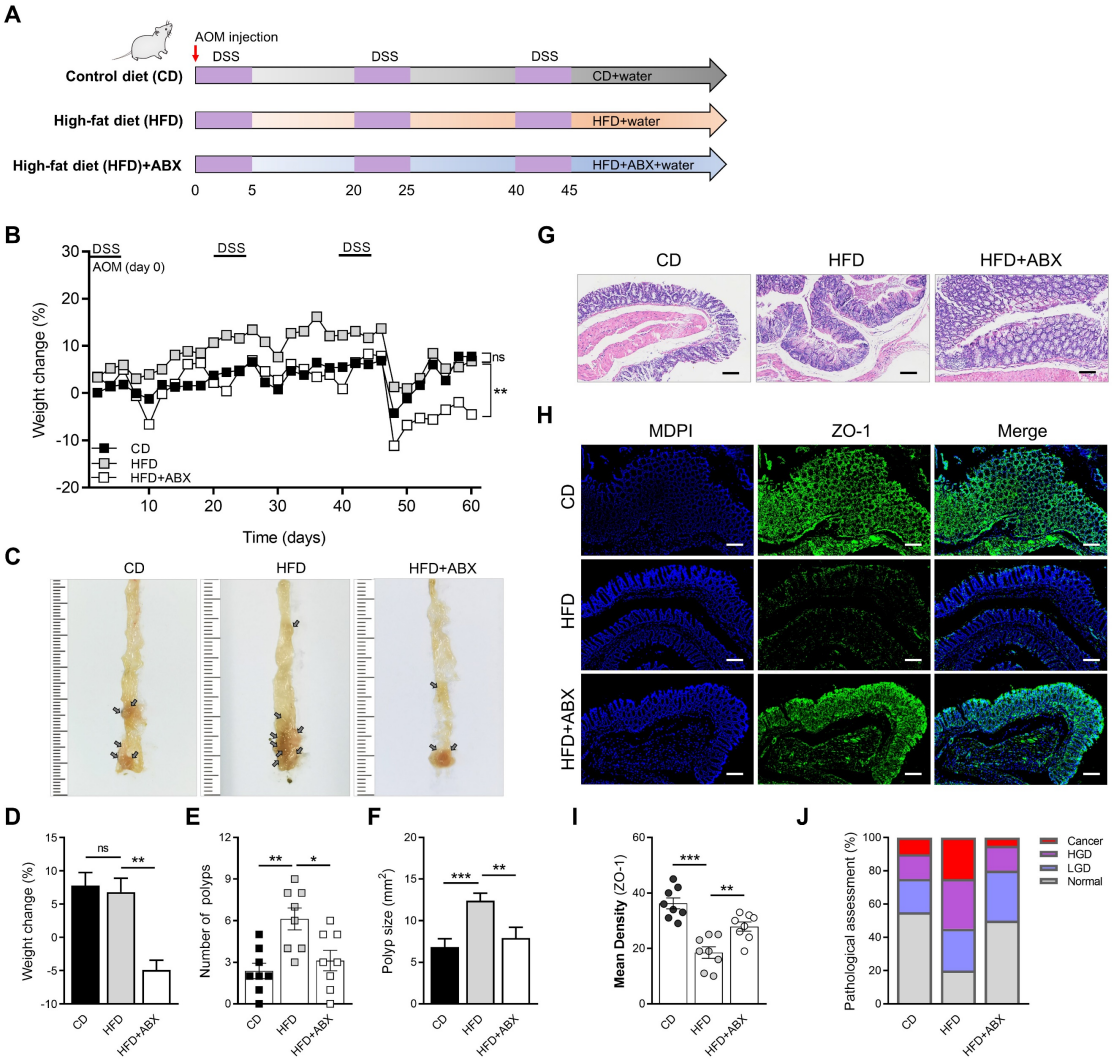
HFD promotes CAC development depending on the gut microbiota. (A) Experimental design for dietary and antibiotics treatment in a CAC mouse model. (B) Weight change in CD-fed, HFD-fed, and ABX-treated HFD-fed mice before sacrifice. (C) Macroscopic polyps (arrows) were identified in the distal and mid colons. (D) Day-60 weight alteration in CD-fed, HFD-fed, and ABX-treated HFD-fed mice before sacrifice. (E-F) The number (E) and maximal cross-sectional area (F) of macroscopic polyps were quantified. (G) Representative pictures of H&E staining in each group. Scale bar = 100 μm. (H) Immunofluorescence staining for ZO-1 in mouse colons. (I) Quantitative analysis of ZO-1 index. (J) Quantitative analysis of pathologic score was calculated according to the following criteria: 0, normal; 1, LGD; 2, HGD; and 3, carcinoma. H&E, hematoxylin and eosin; LGD, low-grade dysplasia; HGD, high-grade dysplasia. CD-fed, n = 8; HFD-fed, n = 8; and ABX-treated HFD-fed, n = 8. ns, no significance; *, *P* < 0.05; **, *P* < 0.01; ***, *P* < 0.001.

To evaluate the effect of HFD on intestinal inflammation in mice and whether it influenced CAC progression, we constructed a CAC model using AOM/DSS and divided mice into HFD and CD groups. To ascertain the role of microbiota alteration in HFD-associated colonic inflammation and CAC, we added the HFD combined with ABX group (Figure 1A). The weight of mice in the HFD group increased significantly in the early stage because of the effect of HFD, but the weight of mice in the later stage decreased obviously (Figure 1B). The significant weight loss in HFD group may be related to the level of intestinal inflammation and tumor growth in mice at the later stage of the experiment. Meanwhile, there were significant differences in colon length, number of colon polyps, and polyp size between mice in the CD and HFD groups (Figure 1C-F, Figure S1A-B). However, when HFD mice were treated with ABX, the changes in colon length, number of colon polyps and polyp size caused by HFD were reversed.

Subsequently, the entire colon was stained with H&E (Figure 1G). The histological activity was then evaluated via individual assessments of inflammation, crypt atrophy, hyperplasia, dysplasia, and the area of inflammation. AOM/DSS-treated HFD mice had significantly enhanced histological activity compared with CD mice, substantiated by a significant increase in inflammation, hyperplasia, and dysplasia, with a greater affected area in HFD mice than in CD mice (Figure S1C-F). Some of these lesions were further characterized as adenocarcinomas in HFD mice.

We then detected the expressions of ZO-1 and Occludin in the entire colon of mice by immunofluorescence staining, and found that the expressions of ZO-1 and Occludin in the HFD group were significantly reduced (Figure 1H-I, Figure S2A-B). Combined with the results of H&E staining, quantitative analysis of pathological score was calculated. The proportion of high-grade dysplasia and low-grade dysplasia in the HFD group was significantly elevated (Figure 1J). However, ABX treatment reversed this effect in mice treated with HFD, further suggesting that HFD may cause disruption of intestinal epithelial cell connectivity and increased inflammation by regulating the gut microbiome.

### Gut microbiota composition is altered by HFD

**Figure 2:**
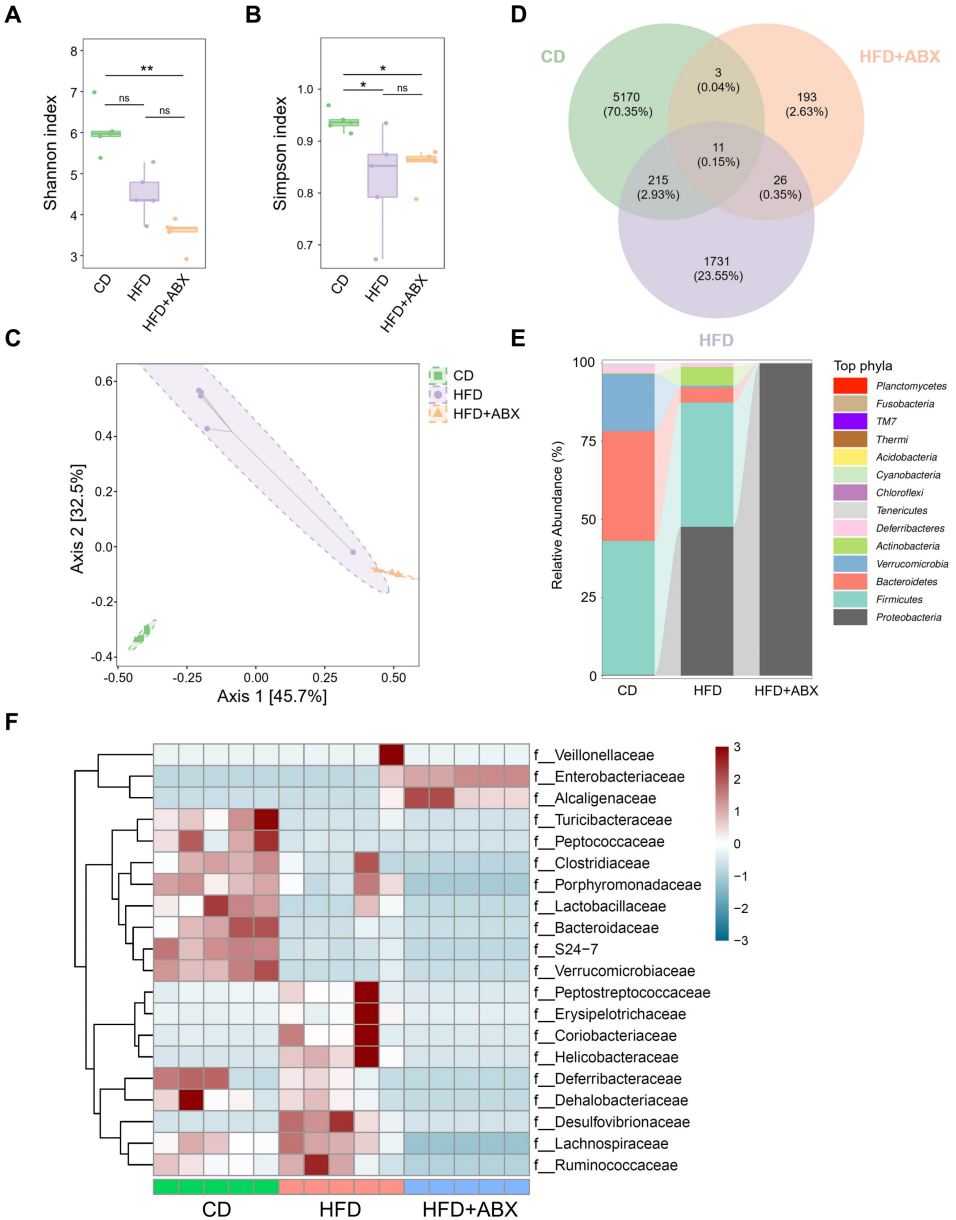
The effects of HFD were correlated with the gut microbiota. (A-B) 16S-rRNA sequencing analysis of fecal samples from CD-fed, HFD-fed, and ABX-treated HFD-fed mice. Shannon (A) and Simpson (B) index analysis in each group. (C) PCoA plot from CD-fed, HFD-fed, and ABX-treated HFD-fed mouse groups based on the Bray-Curtis distance. (D) The ASV/OTU Venn diagram showing the intersection of the three groups. (E) Sankey diagram showing the relative abundance of TOP phyla. (F) Bacterial taxa observed in CD-fed, HFD-fed, and ABX-treated HFD-fed mice, shown at the family level. CD-fed, n = 5; HFD-fed, n = 5; and ABX-treated HFD-fed, n = 5. ns, no significance; *, *P* < 0.05; **, *P* < 0.01.

To investigate whether gut microbiota mediates the promotion of CAC progression by HFD, fecal samples from CD, HFD and HFD combined with ABX groups were analyzed by 16S-rRNA sequencing. In the sequencing analysis, there was no significant difference in the Shannon index values of fecal samples between HFD and CD groups (*P* > 0.05, Figure 2A). In contrast, the Simpson index in the CD group was significantly higher than that in the HFD group (*P* < 0.05, Figure 2B). Moreover, Bray-Curtis distance-based PCoA analysis indicated that the clustering of gut microbiota in the HFD group was significantly different from that in the CD group and HFD combined with ABX group (Figure 2C). Then, a Venn diagram was drawn using the results of ASV/OTU abundance analysis to assess the common or unique microbiome species between CD and HFD groups. The number of microbiome members in each set was counted according to whether they were present in each group. The number of microbiome members in the HFD group was significantly lower than that in the CD group (Figure 2D).

Further analysis showed that although *Firmicutes* was the dominant phylum in CD and HFD groups at the Phyla level, compared with the CD group, the HFD group showed reduced abundance of *Verrucomicrobia* and *Bacteroidetes*, and increased abundance of *Actinobacteria* and *Proteobacteria* (Figure 2E). Taxonomic representation was summarized at the family level (Figure 2F). ABX treatment did induce dysbiosis of gram-positive and -negative bacteria. Compared with the CD, HFD also caused significant differences in gut microbiota abundance, among which known butyrate production-related families, *Clostridiaceae* and *Lactobacillaceae*, were markedly decreased in the HFD group 26-28. We also performed a LEfSe analysis of the fecal 16S rRNA gene sequencing data (Figure S3A-B). In the fecal samples, the taxonomic cladogram obtained from this analysis highlighted significant enrichment of several taxa, related in part to butyrate metabolism, in the CD group compared with the HFD and HFD combined with ABX groups. These results suggested that HFD can significantly alter the structure and abundance of gut microbiota compared with CD, which may be potentially associated with CAC progression.

### Effect of FMT from HFD-fed mice on inflammatory responses and CAC progression

**Figure 3:**
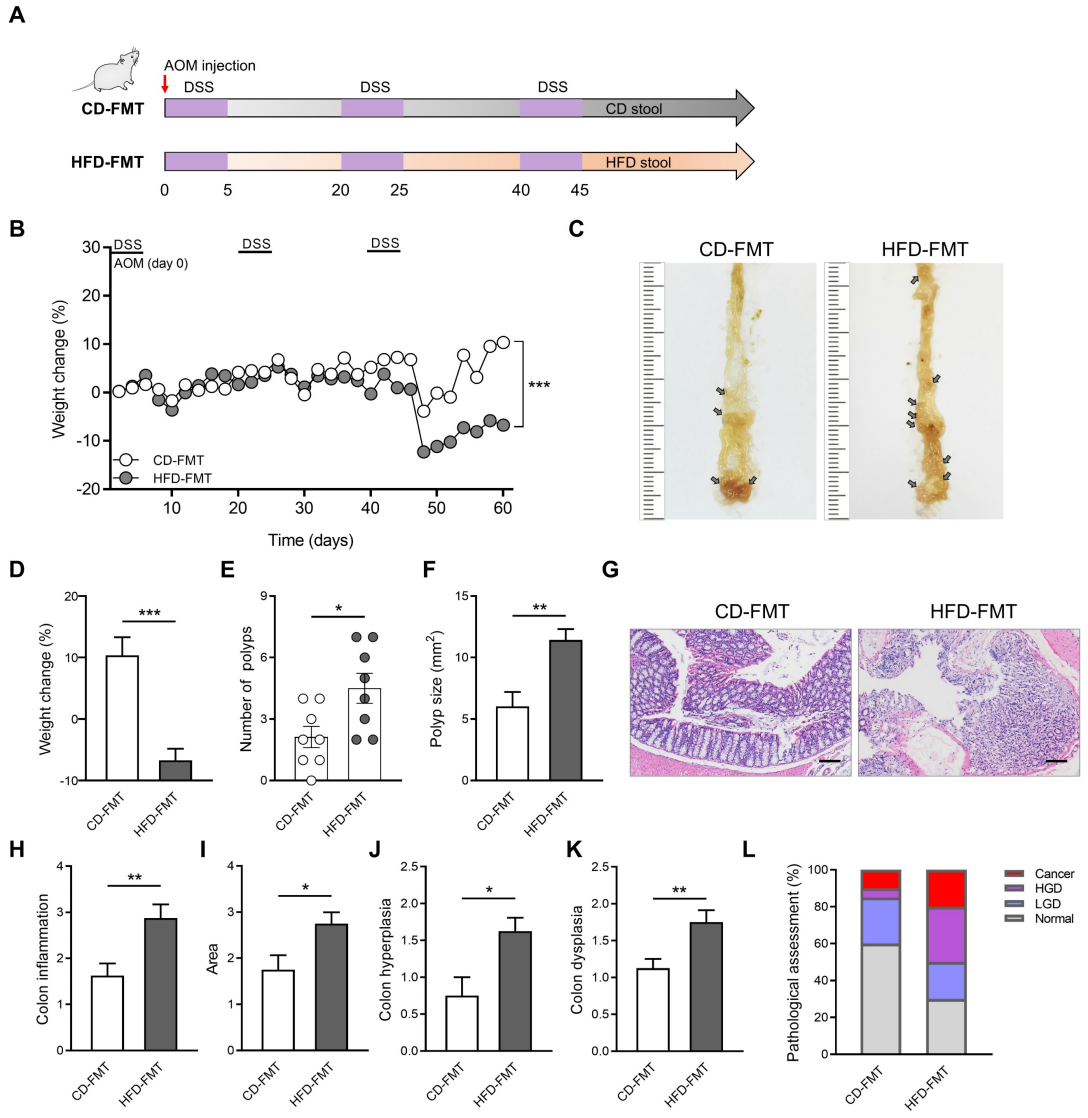
Fecal transplantation with microbiota from HFD-fed mice enhances inflammation-driven CAC. (A) Experimental design for FMT treatment of CD-fed and HFD-fed mice in a CAC mouse model. (B) Weight changes in CD-FMT and HFD-FMT mice before sacrifice. (C) Macroscopic polyps (arrows) were identified in the distal and mid colons. (D) Day-60 weight alterations in CD-FMT and HFD-FMT mice before sacrifice. (E-F) The number (E) and maximal cross-sectional area (F) of macroscopic polyps were quantified. (G) Representative pictures of H&E staining in each group. Scale bar = 100 μm. (H-K) Histopathological analysis of colon inflammation (H), and area associated with disease (I), hyperplasia (J), and dysplasia (K) in CD-FMT and HFD-FMT mice. (L) Quantitative analysis of pathological score was calculated according to the following criteria: 0, normal; 1, LGD; 2, HGD; and 3, carcinoma. H&E, hematoxylin and eosin; LGD, low-grade dysplasia; HGD, high-grade dysplasia. CD-FMT, n = 8; HFD-FMT, n = 8. *, *P* < 0.05; **, *P* < 0.01; ***, *P* < 0.001.

HFD significantly altered the structure and abundance of gut microbiota in mice. To explore whether the alteration of gut microbiota induced by HFD is related to CAC progression, we performed FMT on the CAC model mice obtained by AOM/DSS treatment. During the construction of the CAC mouse model, fecal samples collected from mice in the CD and HFD groups were colonized by gavage (Figure 3A). Compared with the CD-FMT group mice, the HFD-FMT group mice showed significantly reduced weight (Figure 3B). The rapid progression of tumors in the HFD-FMT group may be an important reason for the obvious weight loss in the late experimental period. Moreover, the number of colon polyps and polyp size of mice were more serious and the colon length is significantly shortened in HFD-FMT group compared with the CD-FMT group (Figure 3C-F, Figure S4A-B).

The entire colon was stained with H&E, and histological activity was evaluated (Figure 3G). HFD-FMT mice had significantly enhanced histologic activity compared with CD-FMT mice, substantiated by a significant increase in inflammation, hyperplasia, and dysplasia with a greater affected area in HFD-FMT mice than in CD-FMT mice (Figure 3H-K). For assessing the H&E staining results, quantitative analysis of the pathological score was performed. The proportion of high-grade dysplasia and low-grade dysplasia in the HFD-FMT group significantly elevated (Figure 3L). It suggested that HFD promotes tumorigenesis of CAC by regulating the structure and abundance of gut microbiome.

### Butyrate metabolism mediates HFD-induced colonic inflammatory injury and colitis-associated tumorigenesis

**Figure 4:**
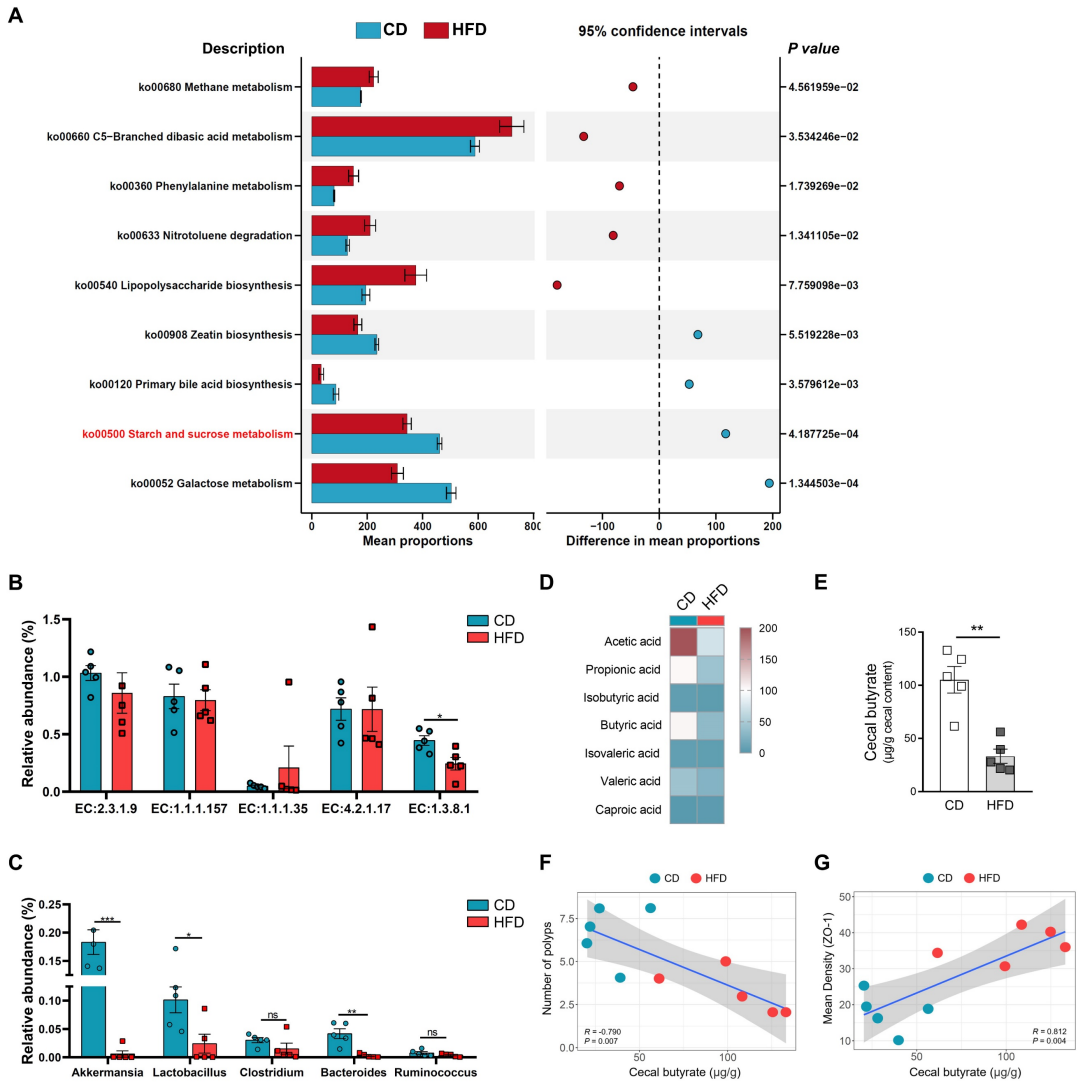
HFD inhibits butyrate-producing bacteria to limit butyrate synthesis and secretion. (A) Prediction of changed butyrate production-associated KEGG pathways using Tax4fun analysis based on the SILVA database. Extended error bar plots illustrate differential representation by mean proportion and their differences based on KEGG orthologue groups, ranked by respective effect size of 0.5 thresholds in the Statistical Analysis of Metagenomic Profiles software package. Bar plots on the left side display the mean proportion of each KEGG pathway. Dot plot analysis on the right shows the differences in mean proportions between the two indicated groups using *P* values. (B) Butyrate synthesis-related enzymes. (C) Relative abundance of butyrate-producing microbiota and probiotics in fecal samples. (D) Heatmap of fecal SCFAs concentrations. (E) Differences in fecal butyrate concentrations between CD and HFD mice. (F) Assessment of the correlation between cecal butyrate and number of polyps. (G) Assessment of the correlation between cecal butyrate and ZO-1 expression level in the intestinal mucosa. n = 5 in each group. ns, no significance. *, *P* < 0.05; **, *P* < 0.01; ***, *P* < 0.001.

After investigating whether HFD promotes the tumorigenesis of CAC by regulating the structure and abundance of the gut microbiome, we found that the analysis highlighted significant enrichment in several taxa, related in part to butyrate metabolism. To clarify the underlying mechanisms for the association between the alteration of gut microbiota caused by HFD and butyrate metabolism, 16S-rRNA gene-predicted functional profiles, associated with butyrate metabolism, were analyzed using Tax4fun based on the SILVA database (Figure 4A). Comparisons of the Kyoto Encyclopedia of Genes and Genomes (KEGG) orthologues for pathway enrichment showed a decrease in the pathways related to starch and sucrose metabolism (ko00500), which are associated with butyrate production, following HFD treatment 29.

Butyrate metabolism and secretion are closely related to the level of intestinal inflammation, and intestinal inflammation is one of the most important factors affecting CAC progression 30, 31. To assess the influence HFD exerts on butyrate metabolism, the relative abundances of butyrate synthesis-related enzymes were analyzed 32.

EC:1.3.8.1 was obviously inhibited in the HFD group compared with the CD group (Figure 4B). Subsequently, we also found that the relative abundances of *Bacteroides*, *Lactobacillus,* and *Akkermansia* were significantly enriched in the HFD group (Figure 4C). *Bacteroides*, *Lactobacillus,* and *Akkermansia* were closely associated with the synthesis of butyrate and intestinal health 26, 33, 34. Based on the analysis of butyrate metabolism-related enzymes and microbiota, we further evaluated the contents of butyrate in the guts of mice. Compared with the CD mice, the HFD mice showed significantly reduced SCFAs content in the colon, especially that of butyrate (*P* < 0.01, Figure 4D-E). These results suggest that HFD inhibits the synthesis and secretion of SCFAs, particularly butyrate.

HFD reduces the abundance of butyrate-producing bacteria, and inhibits the synthesis and secretion of butyrate. However, whether HFD affects the tumorigenesis of CAC in mice and its potential mechanisms need to be further elucidated. Therefore, we evaluated the correlation between cecal butyrate levels and number of polyps. The concentrations of cecal butyrate were negatively correlated with the number of polyps (*P* = 0.007, Figure 4F). Further, there was a significant positive correlation between cecal butyrate and ZO-1 expression (*P* = 0.004, Figure 4G). This indicated that HFD exacerbated intestinal inflammation and accelerated the progression of CAC by inhibiting butyrate synthesis and secretion.

### Butyrate regulates HFD-induced colonic pro-inflammatory macrophage infiltration and M1/M2 polarization

**Figure 5:**
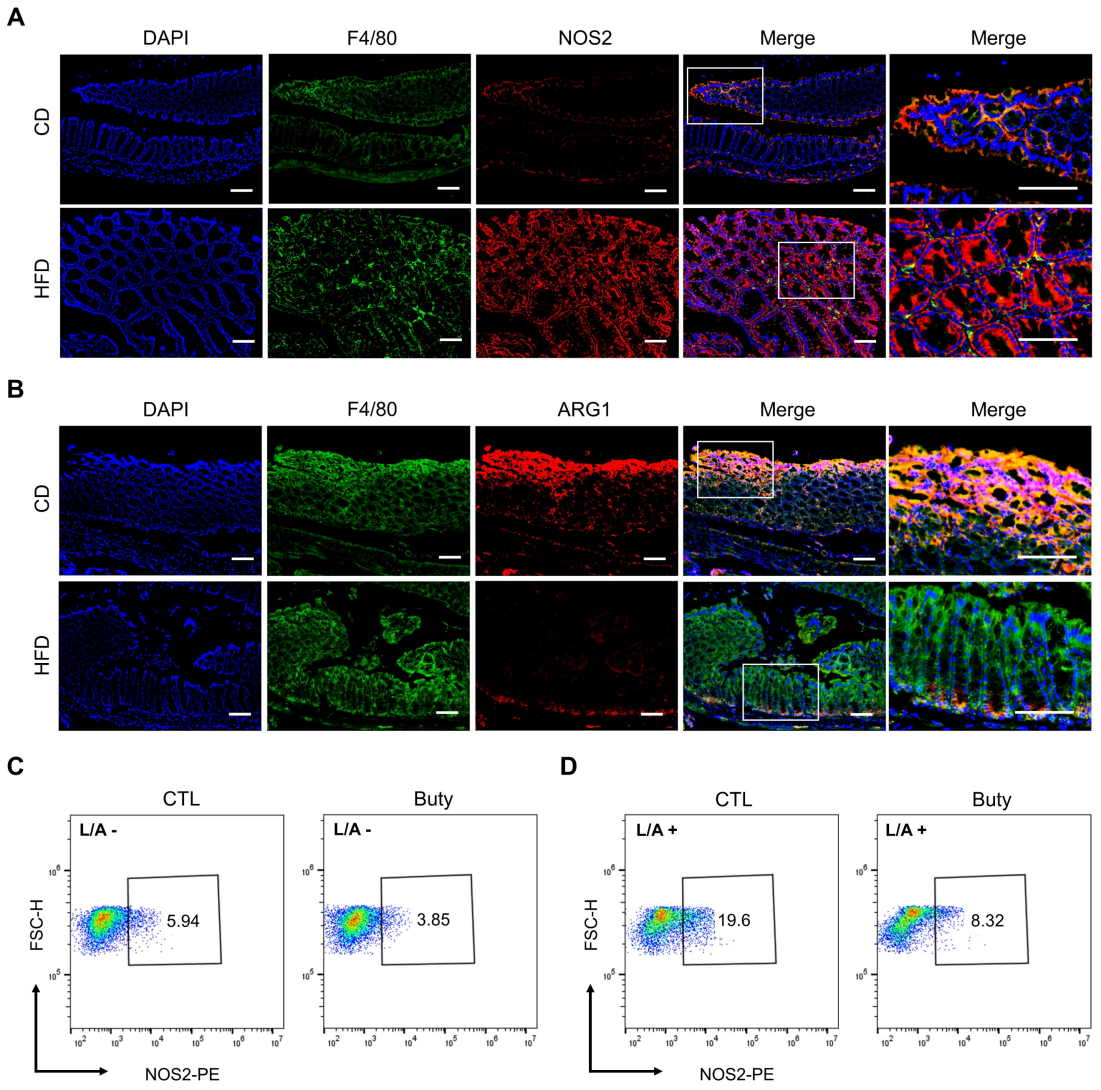
HFD promotes macrophage polarization by regulating butyrate metabolism. (A) Representative images of immunofluorescence staining for macrophage marker F4/80 (green); M1 marker, NOS2 (red); and F4/80^+^NOS2^+^ cells (yellow, merge). (B) Representative images of immunofluorescence staining for macrophage marker F4/80 (green); M2 marker, ARG1 (red); and F4/80^+^ARG1^+^ cells (yellow, merge). Cell nuclei, DAPI (blue). Scale bars = 100 μm. (C-D) Macrophage M1 polarization investigation after butyrate treatment for 24 h, without (C) or with (D) LPS/ATP stimulation. Buty, butyrate. L/A, LPS/ATP.

Owing to aggravative inflammation in the gut regulated by M1 macrophage polarization and loss of gut microbial homeostasis, M1/M2 polarization of macrophages plays an important role in intestinal inflammation 35. To assess whether the effect of HFD was associated with M1/M2 macrophage polarization in mouse colon tissue, we performed immunofluorescence staining for the colocalization of the macrophage marker F4/80 with the M1 macrophage marker NOS2 (Figure 5A) or the M2 macrophage marker ARG1 (Figure 5B). The results showed that F4/80^+^NOS2^+^ macrophages were markedly elevated in the HFD group, and the abundance of F4/80^+^ARG1^+^ macrophages was obviously reduced in the colon tissues in the HFD group compared with CD group.

In addition, the effect of butyrate on M1 macrophage polarization was investigated *in vitro*. In the *RAW264.7* macrophage cell line treated with butyrate, the results revealed that the macrophage M1 polarization in the butyrate-treated group was moderately lower than that in the control group (Figure 5C). Meanwhile, to assess the pro-inflammatory process of macrophages, LPS and ATP were used to induce *RAW264.7* M1 polarization. The results indicated that butyrate significantly inhibited the M1 polarization of macrophages (Figure 5D).

### Butyrate blocks accelerated colitis-associated tumorigenesis caused by HFD-FMT

**Figure 6:**
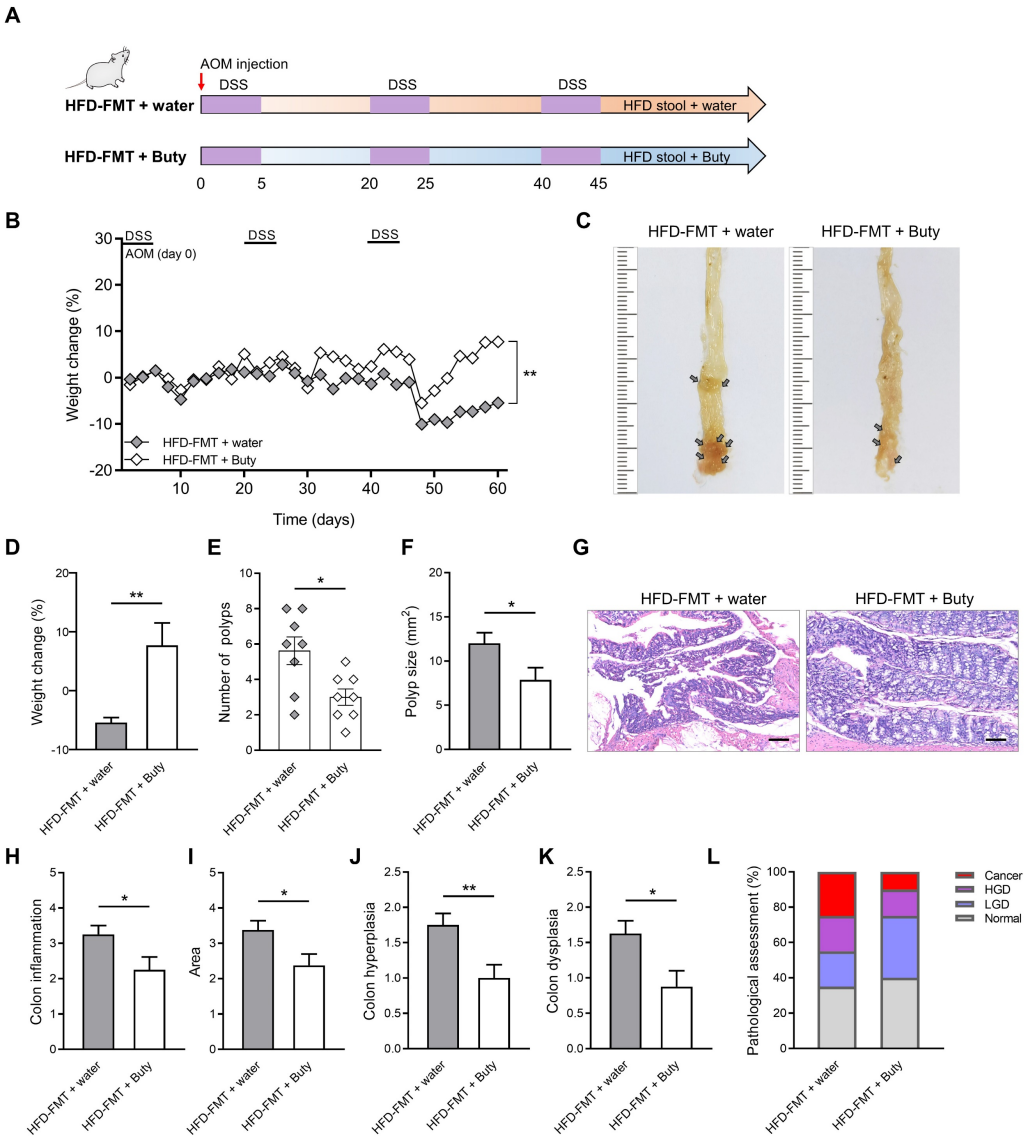
Butyrate reverses the influence of fecal transplantation with microbiota from HFD-fed mice. (A) Experimental design for butyrate treatment of HFD-fed mice with FMT in a CAC mouse model. (B) Weight changes in the water and butyrate treatment with HFD-FMT before sacrifice. (C) Macroscopic polyps (arrows) were identified in the distal and mid colons. (D) Day-60 weight alterations in water and butyrate treatment with HFD-FMT before sacrifice. (E-F) The number (E) and maximal cross-sectional area (F) of macroscopic polyps were quantified. (G) Representative pictures of H&E staining in each group. Scale bar = 100 μm. (H-K) Histopathological analysis of colon inflammation (H), area associated with disease (I), hyperplasia (J), and dysplasia (K) following water and butyrate treatment with HFD-FMT. (L) Quantitative analysis of pathological score was calculated according to the following criteria: 0, normal; 1, LGD; 2, HGD; and 3, carcinoma. H&E, hematoxylin and eosin; LGD, low-grade dysplasia; HGD, high-grade dysplasia. Buty, butyrate. n = 8 in each group. *, *P* < 0.05; **, *P* < 0.01.

Butyrate relieves intestinal inflammation by inhibiting M1 polarization of macrophages. Therefore, the exogenous supplementation of butyrate may be an effective intervention to block the development of HFD-induced CAC. To clarify the effect butyrate exerts on the process of CAC, after performing HFD-FMT, butyrate was added to the drinking water of CAC mice (Figure 6A).

During the construction of the CAC model combined with HFD-FMT, the weight of mice treated with butyrate obviously reversed (Figure 6B). The reason for this may be that butyrate alleviates intestinal inflammation level and interferes with CAC progression. Further evaluation confirmed this hypothesis, and we found butyrate inhibited the number of colon polyps and polyp size of CAC mice, and the colon length was significantly reversed (Figure 6C-F, Figure S5A-B). In addition, butyrate reduced histological activity including inflammation, hyperplasia, and dysplasia with a greater affected area (Figure 6G-K). According to the results of H&E staining, butyrate treatment also significantly decreased the proportion of high-grade dysplasia and low-grade dysplasia (Figure 6L).

### HFD activates NLRP3-associated pro-inflammatory mediator expression by inhibiting butyrate metabolism

**Figure 7:**
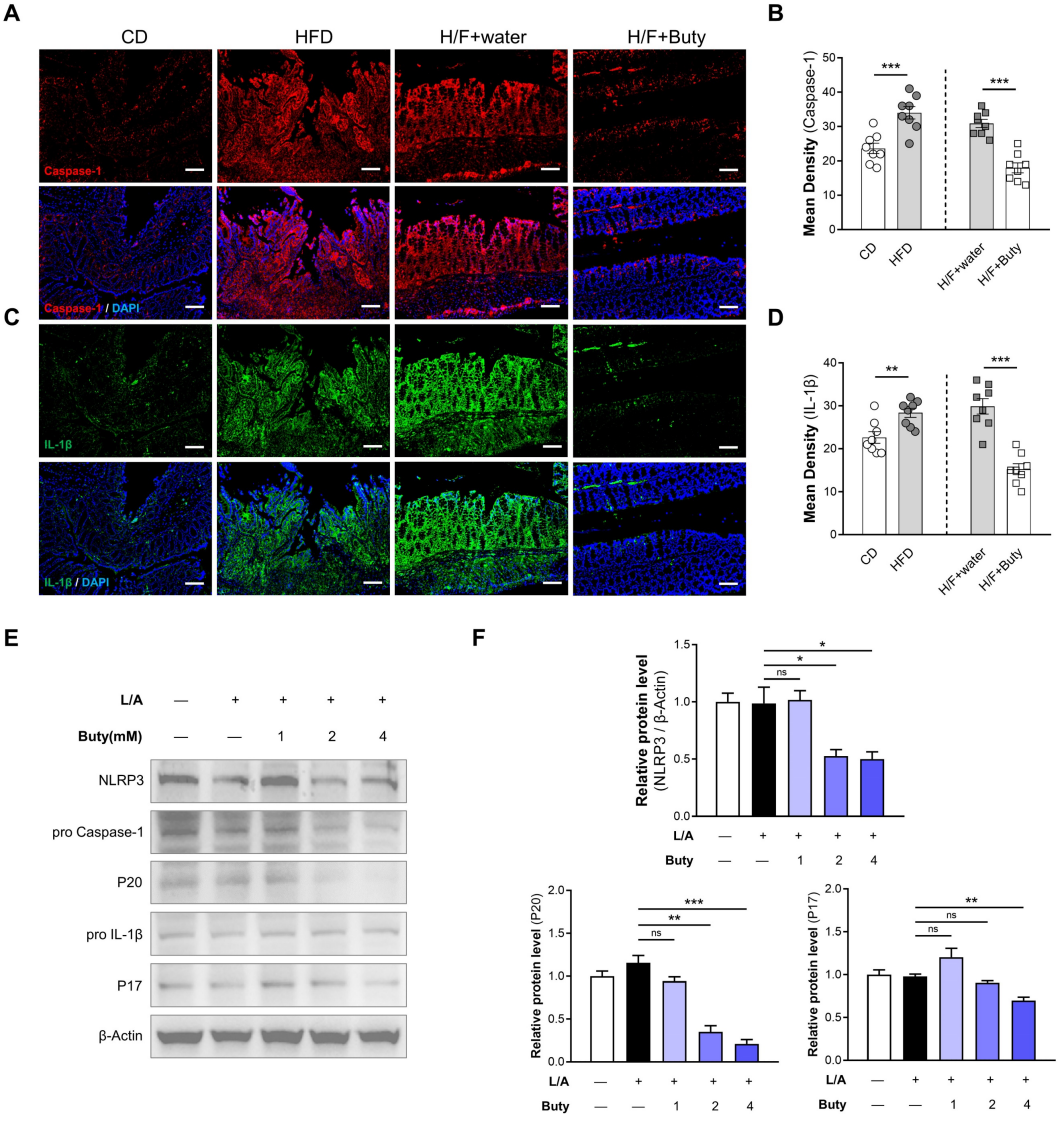
HFD promotes NLRP3-associated pro-inflammatory mediator expression by limiting butyrate synthesis and secretion. (A) Representative immunofluorescence staining of Caspase-1 in colon tissues of mice with CD, HFD, H/F+water, and H/F+butyrate treatment. Scale bars = 100 μm. (B) Quantification of Caspase-1 expression by calculating mean density (integrated density/specimen area). (C) Representative immunofluorescence staining of IL-1β in colon tissues (D) Quantification of IL-1β expression by calculating mean density (integrated density/specimen area). (E) The expression levels of NLRP3, cleaved caspase-1 (P20), and mature IL-1β (P17) were detected after treatment with different concentrations of butyrate. (F) The bands were quantified. H/F, HFD-FMT. Buty, butyrate. L/A, LPS/ATP. ns, no significance; *, *P* < 0.05; **, *P* < 0.01; ***, *P* < 0.001.

The host may maintain tolerance to intestinal microbiota alterations caused by HFD by inhibiting macrophages responsive to commensal bacteria by decreasing proinflammatory effectors via butyrate metabolism. To explore the underlying mechanism, we detected the NLRP3/Caspase-1-associated inflammatory pathway. The results of immunofluorescence showed that in the colon tissues of mice, HFD increases the Caspase-1 and IL-1β expression levels, which are important parameters of NLRP3 inflammasome activation (Figure 7A-D). However, butyrate inhibits the activation of the NLRP3-dependent inflammatory pathway induced by HFD-FMT.

We also found the same phenomena *in vitro* via Western blotting. The NLRP3 expression level was obviously reduced in cells treated with butyrate (Figure 7E-F). Moreover, butyrate treatment showed lower levels of cleaved caspase-1 (P20) and IL-1β (P17) compared with control treatment. Treatment of macrophages with butyrate inhibited the M1 polarization of macrophages, resulting in the downregulation of several pro-inflammatory mediators because of the inhibition of histone deacetylases by butyrate 36, 37. This indicated that HFD activates NLRP3-associated pro-inflammatory mediator expression by inhibiting butyrate metabolism.

## Discussion

Colorectal cancer is usually caused by both genetic and environmental factors. Besides adenoma carcinogenesis, serrated lesion carcinogenesis and other carcinogenic processes, inflammation is one of the widely recognized carcinogenic ways 38, 39. Hence, a particularly aggressive subtype of colorectal cancer is CAC. The intestinal mucosa of patients with IBD is at high risk of developing CAC because of long-term exposure to inflammation. Studies have found that when low-grade inflammation occurs, inflammation of the colon precedes that of other tissues, eventually leading to inflammation of the distal organs 19, 40.

With the advent of genome sequencing and microbiome identification, the diversity of the gut microbiota is becoming clearer. Gut microbiota has been shown to be closely related to the maintenance of health, and alterations in the microbiome have been linked to the occurrence of many diseases 41, 42. The intestinal tract forms a barrier between the body and the contents of the intestinal cavity by a single layer of epithelial cells. Thus, the intestinal immune system must constantly maintain a balance between tolerance toward symbiotic bacteria and immunity against pathogenic bacteria. An imbalance in this equation is one of the important causes of excessive intestinal inflammatory response 1, 33, 43. Although there is evidence that intestinal inflammation associated with HFD is caused by changes in the microbiome and mucosal immune homeostasis, the detailed mechanisms through which intestinal inflammation is triggered remain unclear 1, 3. In this study, a CAC mouse model was constructed to investigate the effect of HFD on CAC tumor progression. The results showed that HFD accelerated the progression of CAC in mice, and it could be reversed by ABX. These results suggest that HFD accelerates CAC depending on gut microbiota and related metabolites.

Based on the finding that HFD promotes CAC progression and is linked to gut microbiota, we performed a prediction of changed KEGG pathways using Tax4fun analysis according to the SILVA database by 16S-rRNA sequencing 29. Our analysis revealed that HFD inhibited the abundance of butyrate-producing bacteria. To further confirm this conclusion, we examined the concentration of SCFAs in the gut of mice treated with HFD, and found that SCFAs synthesis and secretion, those if especially butyrate, were significantly inhibited. SCFAs, as important small molecule metabolites, are a potential medium for gut microbiota to regulate intestinal inflammatory response. There are many SCFAs-producing bacteria in the gut, which produce SCFAs by anaerobic fermentation of dietary fiber 44-46. SCFAs, especially butyrate, act as special nutritional and energy components of the intestinal epithelium, protect the intestinal mucosal barrier, and reduce human inflammation 31, 32. Our study also found that the content of butyrate in the gut of mice was negatively correlated with the number of polyps, but positively correlated with the expression of tight junction protein ZO-1, suggesting that HFD affects the level of intestinal inflammation by regulating the abundance of butyrate-producing bacteria and butyrate synthesis and secretion, in turn accelerating the progression of tumors.

Laminae propria macrophages are important participants in intestinal immune balance, and they can quickly respond to environmental changes 47. Activation and infiltration of macrophages play a central role in colon inflammation. HFD promoted the M1 polarization of macrophages in the process of aggravating intestinal inflammation and accelerating tumor progression. The M1 polarization of macrophages activates the NLRP3-dependent inflammatory pathway and enhances the release of IL-1β, IL-18, and other pro-inflammatory factors, further aggravating the level of intestinal inflammation 37. NLRP3 inflammasome is a polymeric cytoplasmic protein complex that promotes the maturation and release of IL-1β and IL-18 inflammatory cytokines, leading to systemic inflammation. NLRP3 recruits an apoptosis-related speckle like protein of the junction molecule containing the Caspase-recruitment domain, which in turn binds to Caspase-1, leading to autocatalytic processing and activation 48-50. NLRP3 inflammasomes are closely associated with the regulation of homeostasis and maintenance of immune response in the gut. It suggests that HFD plays a crucial role in proinflammatory macrophage infiltration and tumorigenesis of IBD-related colonic tumors.

Gut microbiota and their metabolites actively participate in various physiological and pathological processes in the intestine. Of these, butyrate-producing bacteria are an extremely important part 51, 52. Our study explored the effect of HFD on intestinal butyrate-producing bacteria and butyrate metabolism. To clarify the regulatory role of butyrate on intestinal inflammation, we conducted relevant experiments *in vivo* and *in vitro*. The *in vivo* experiments indicated that butyrate could reverse the aggravation of intestinal inflammation and the accelerated tumor growth induced by HFD-FMT, and the NLRP3/Caspase-1 pathway in the colon activated by HFD-FMT was also significantly inhibited by butyrate. To verify the effect of butyrate on macrophages *in vitro*, macrophages were treated with LPS combined with butyrate to detect the M1 polarization level and NLRP3/Caspase-1 pathway expression, and the results were consistent with those of the *in vivo* experiments.

The effects of alterations in the gut microbiota abundance, the level on SCFAs metabolism, and the intestinal microenvironment are a comprehensive and complex biological process, which is affected by many factors 53, 54. SCFAs regulate immune cells in this process, reduce the excessive immune response caused by colonic symbiote changes, and maintain homeostasis of the internal microenvironment. Butyrate is believed to inhibit secondary response genes by recruiting Mi-2/NuRD inhibitor complexes, which enable Mi-2 to inhibit chromatin remodeling of secondary response genes. Butyrate inhibits these pro-inflammatory mediators at the transcriptional level, resulting in reduced recruitment of NOS2, IL-6, and IL-12b promoters by pol II and S5P 55-57. These findings suggest the role of butyrate in immune regulation. HFD inhibits the abundance of various butyrate-producing bacteria and interferes with the synthesis and secretion of butyrate, which is an important reason for the aggravation of intestinal inflammation and development of CAC.

However, the role of butyrate in NLRP3 inflammasome regulation is controversial. Our previous studies have clarified that butyrate regulates the immune response of intestinal macrophages and down-regulates pro-inflammatory mediators by inhibiting histone deacetylase 31, 33. In addition, butyrate has been believed to protect intestinal epithelial cells from *Clostridium difficile* toxin-induced damage by stabilizing HIF-1, thus alleviating local inflammatory response 58, 59. However, other studies have reported a different story. They have linked the worsening of colitis to the enrichment of butyric acid-producing bacteria and increased butyric acid levels in the cecum. It was also assessed that excessive butyrate may damage the intestine by inhibiting the proliferation of epithelial stem/progenitor cells in the colon 25, 60.

Researchers suspect that the different effects of butyrate depend on the specific microenvironment, but the mechanism is unclear and needs further study. Although inhibition of M1 polarization of macrophages by butyrate may reverse the increased colon inflammation caused by HFD, it cannot be ruled out that chronic exposure of colon epithelial cells to relatively high levels of butyrate can induce epithelial barrier damage 25, 60, 61. Under normal diet conditions, the concentration of butyrate is relatively low in the gut and does not cause significant epithelial barrier damage. However, attention should be paid to avoiding excessively high level of butyrate when considering interventions with exogenous butyrate treatments for intestinal mucosal damage caused by HFD.

Our study also had some limitations. In this study, we investigated the promoting effect of HFD on CAC and its potential mechanism *in vivo* and *in vitro*. Future prospective studies, including detailed CAC assessments, and glycolipid metabolism analysis could demonstrate and support our hypothesis. In the future, multivariate analysis of clinical data and demographics, such as modeling including covariates, can be performed to assess the association between HFD and CAC.

## Conclusions

**Figure 8:**
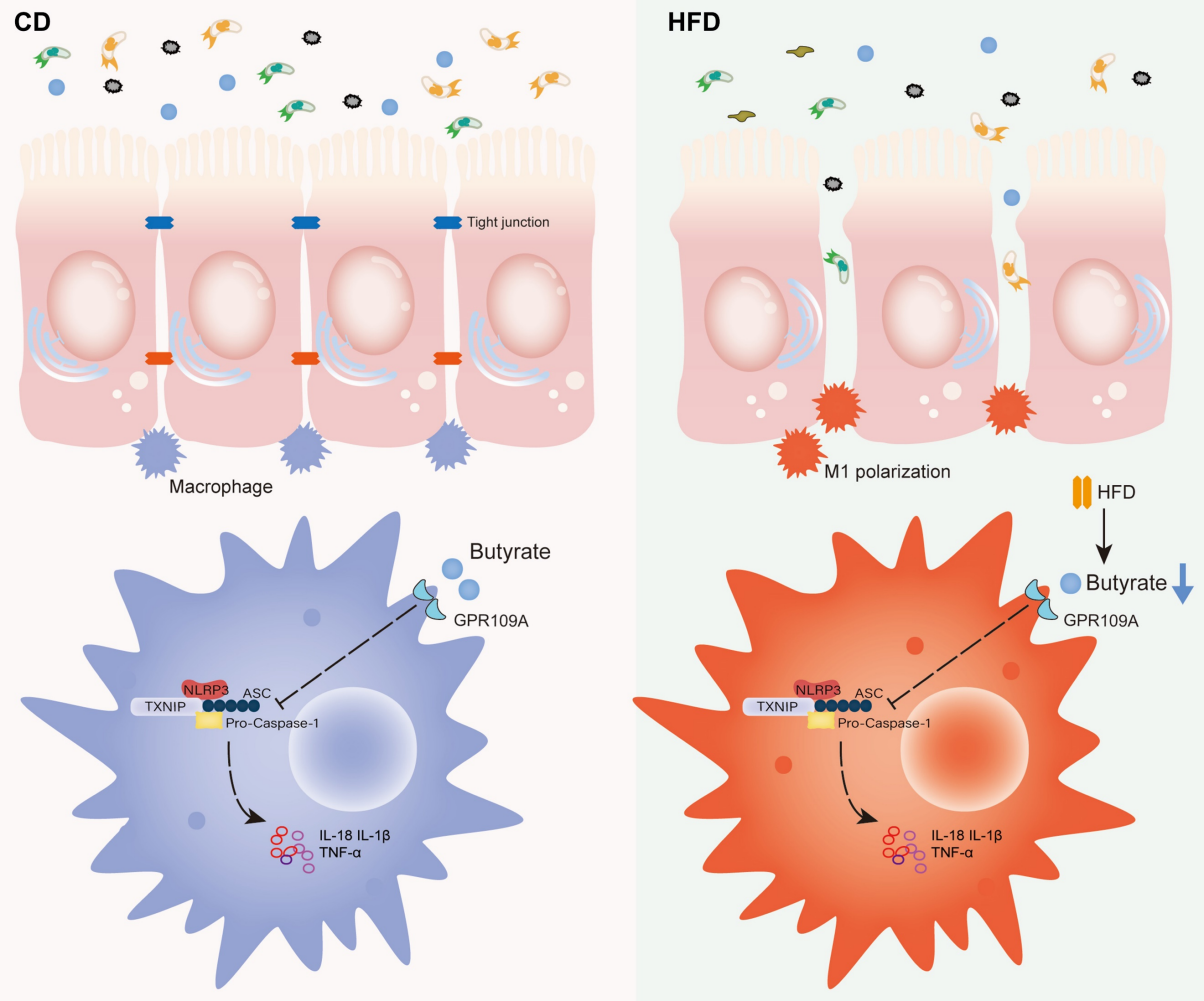
Proposed model and the underlying mechanism through which HFD regulates the abundance of the gut microbiota to change the concentration of butyrate, thereby affecting intestinal inflammation and promoting inflammation-driven CAC.

In summary, HFD promotes the M1 polarization of gut macrophages and aggravates intestinal inflammation by inhibiting the abundance of butyrate-producing bacteria and interfering with butyrate metabolism. It ultimately accelerates the occurrence and development of CAC (Figure 8). For IBD patients, restriction of HFD is a qualified choice to regulate gut microbiota to produce microbial metabolite butyrate and play an anti-inflammatory role. Meanwhile, the FMT of butyrate-producing bacteria and adding exogenous butyrate are feasible new treatment strategies for IBD and CAC, and have good prospect of clinical application.

## Supplementary Material

Supplementary figures.

## Abbreviations

CAC: Colitis associated cancer
HFD: High-fat diet
FMT: Fecal microbiota transplantation
ABX: Broad-spectrum antibiotics
SCFAs: Short-chain fatty acids
IBD: Inflammatory bowel disease
UC: Ulcerative colitis
SPF: Specific pathogen-free
AOM: Azoxymethane
CD: Control diet
H&E: Hematoxylin and eosin
GC-MS: Gas chromatography-mass spectroscopy
ATP: Adenosine triphosphate
LPS: Lipopolysaccharide
SDS-PAGE: Sodium dodecyl sulfate-polyacrylamide gel electrophoresis
PVDF: Polyvinylidene fluoride
KEGG: Kyoto Encyclopedia of Genes and Genomes
