# Supplementary material for: High-fat diet promotes colitis-associated tumorigenesis by altering gut microbial butyrate metabolism
Source: Int J Biol Sci. 2023 Sep 25;19(15):5004–19. doi: 10.7150/ijbs.86717 (PMC10539701; doi:10.7150/ijbs.86717)
Supplement: Supplementary file 1 — Supplementary figures. [file ijbsv19p5004s1.pdf]

Supplementary Materials

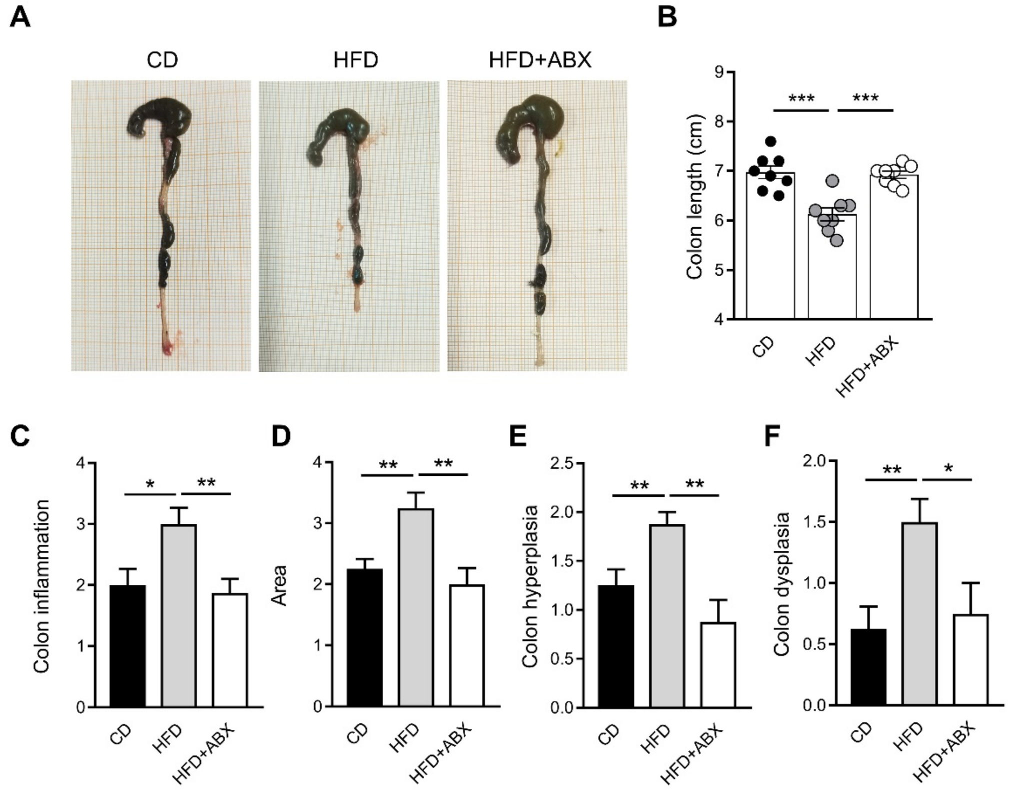

Supplementary Figure S1. Effects of HFD on intestinal inflammation in mice. (A) Differences in the whole colon in CD-fed, HFD-fed, and ABX-treated HFD-fed mice. (B) Total colon length in the mice in each group. (C-F) Histopathological analysis of colon inflammation (C), area associated with disease (D), hyperplasia (E), and dysplasia (F) in CD-fed, HFD-fed, and ABX-treated HFD-fed mice. CD-fed, n = 8; HFD-fed, n = 8; and ABX-treated HFD-fed, n = 8. *, $P < 0.05$; **, $P < 0.01$; ***, $P < 0.001$.

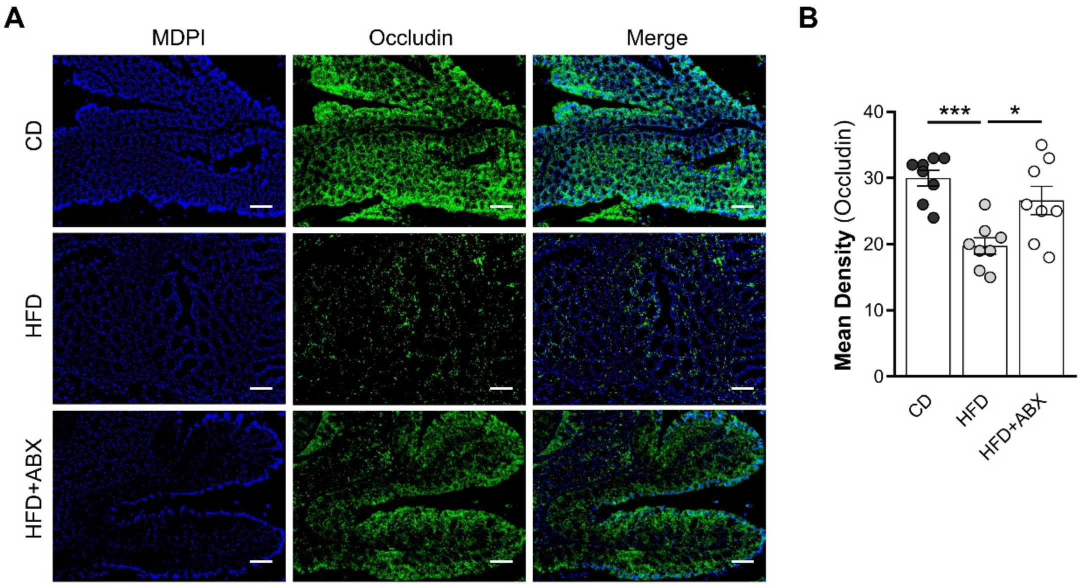

Supplementary Figure S2. High-fat diet affects intestinal mucosal tightness. (A) Immunofluorescence staining of mice colons for Occludin. (B) Quantitative analysis of Occludin index. CD-fed, n = 8; HFD-fed, n = 8; and ABX-treated HFD-fed, n = 8. *, $P < 0.05$; ***, $P < 0.001$.

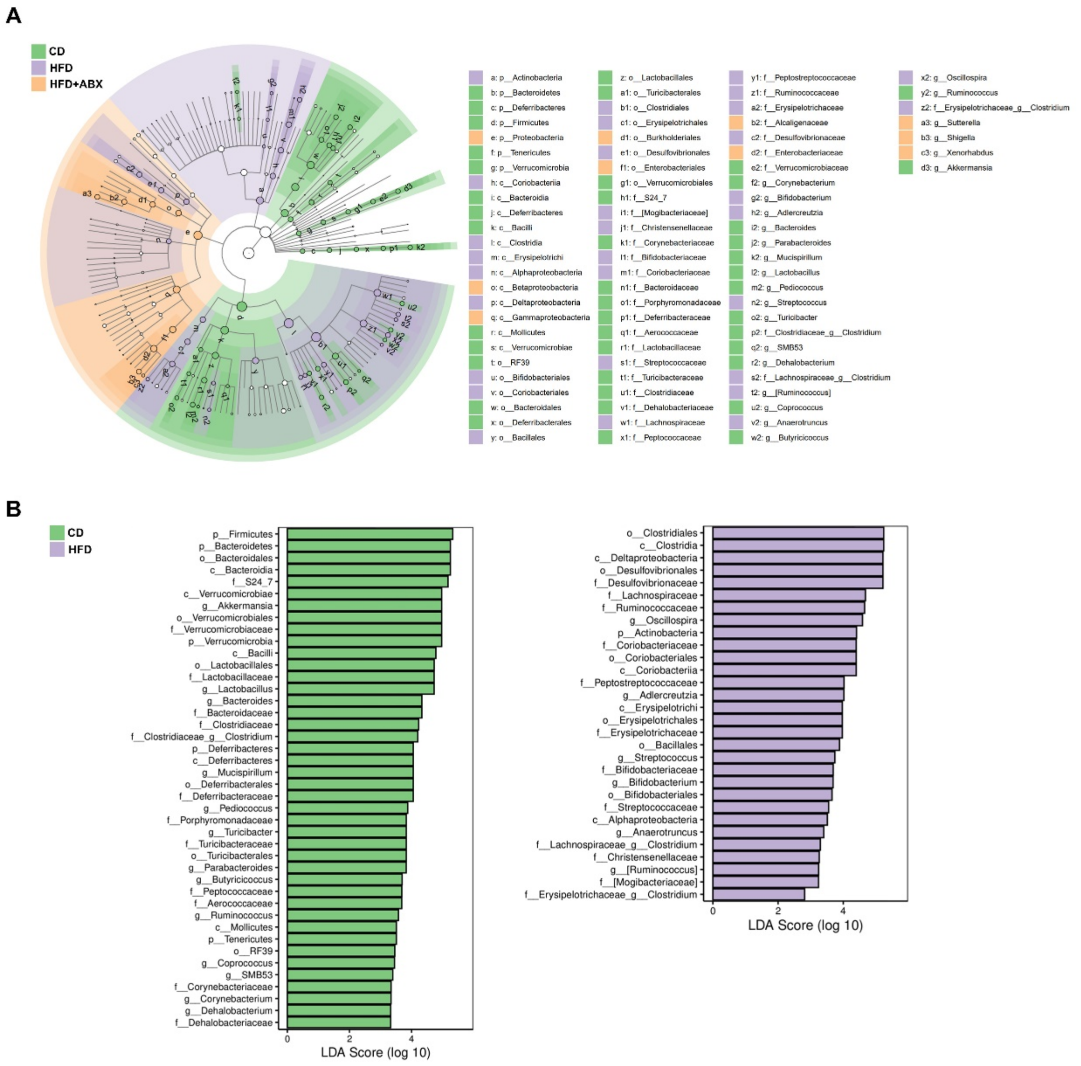

Supplementary Figure S3. Effects of HFD on intestinal flora distribution. (A-B) LEfSe analysis of gut microbiota alterations caused by HFD (n=5 in each group).

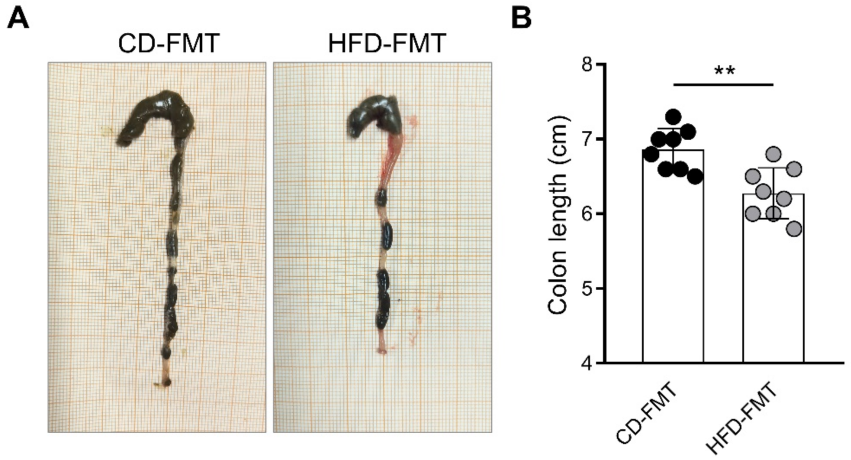

Supplementary Figure S4. Fecal transplantation with microbiota from HFD-fed mice enhances intestinal inflammation. (A) Differences in the whole colon in CD-FMT and HFD-FMT. (B) Total colon length in the mice in each group. CD-FMT, n = 8; HFD-FMT, n = 8. **, $P < 0.01$.

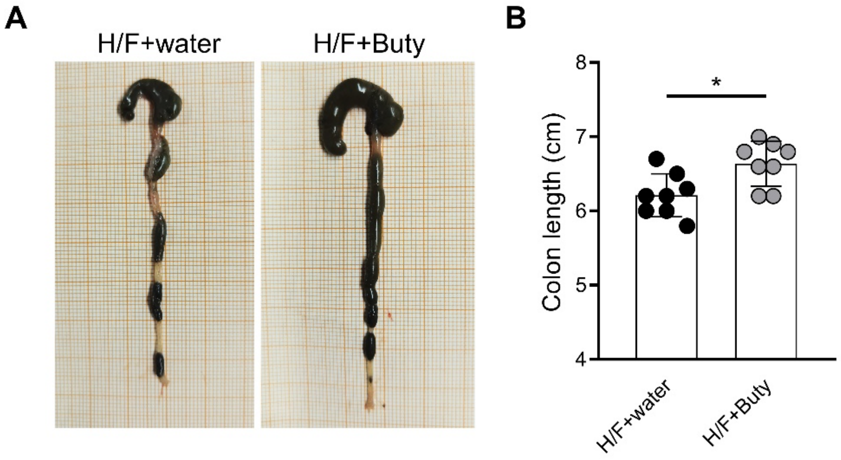

Supplementary Figure S5. Butyrate alleviates inflammation levels increased by fecal transplantation with microbiota from HFD-fed mice. (A) Differences in the whole colon in H/F+water and H/F+butyrate. (B) Total colon length in the mice in each group. H/F, HFD-FMT. Buty, butyrate. H/F+water, n = 8; H/F+butyrate, n = 8. *, $P < 0.05$.
